# Background Activity Findings in End-Stage Renal Disease With and Without Comorbid Diabetes: An Electroencephalogram Study

**DOI:** 10.3389/fnhum.2021.741446

**Published:** 2021-10-08

**Authors:** Tirapoot Jatupornpoonsub, Paramat Thimachai, Ouppatham Supasyndh, Yodchanan Wongsawat

**Affiliations:** ^1^Brain Computer Interface Laboratory, Department of Biomedical Engineering, Faculty of Engineering, Mahidol University, Salaya, Thailand; ^2^Nephrology Division, Department of Medicine, Phramongkutklao Hospital, Bangkok, Thailand

**Keywords:** QEEG, diabetes, end-stage renal disease, renal failure, encephalopathy

## Abstract

Renal failure and diabetes can induce cerebral complications, including encephalopathy, for which attentional and cognitive impairment are common symptoms. It is possible that renal failure with comorbid diabetes may induce more severe encephalopathy due to multiple pathogenic mechanisms. This concept was supported by the main findings of this study, which showed that EEG background activity between end-stage renal disease with and without comorbid diabetes was significantly different in relative power of delta in the eyes-open condition in frontoparietal regions; theta in the eyes-closed condition in all regions; beta in the parieto-occipital regions in both eye conditions; the delta/theta ratio in both eye conditions in frontoparietal regions; and the theta/beta ratio in all regions in the eyes-closed condition. These findings may increase awareness of comorbid cerebral complications in clinical practice. Moreover, the delta/theta ratio is recommended as an optimal feature to possibly determine the severity of encephalopathy.

## 1. Introduction

End-stage renal disease (ESRD), a failure of regulatory and excretory renal function, has been known to directly cause uremic toxin accumulation, blood-brain barrier damage, and serum electrolyte derangement, among other problems (Chillon et al., 2016; Jabbari and Vaziri, 2018; Hamed, 2019). These effects consequently induced many brain complications. In fact, eighty percent of ESRD patients have explicit cerebral complications, although they routinely receive either machinery hemodialysis (MHD) or peritoneal dialysis (PD) (Hamed, 2019). Cognitive deficit and uremic encephalopathy are two common forms of cerebral dysfunction that occur in ESRD (Rayi and Mandalaneni, 2020). Diabetes mellitus (DM), an abnormality of serum glucose regulation, can also induce diabetic encephalopathy and cognitive impairment. These are similar to the cerebral complications of ESRD (Cai et al., 2011; Rayi and Mandalaneni, 2020). In particular, encephalopathy, usually referred to as diffuse cerebral dysfunction, was suggested to be associated with a slowing of background activity in both delta and theta frequency bands on an electroencephalogram (EEG) (Kaplan and Rossetti, 2011; Sutter et al., 2013; Demir et al., 2014; Rayi and Mandalaneni, 2020). The EEG background activity can be observed in both the time domain and frequency domain. Traditionally, time-domain EEGs in the anterior-posterior bipolar montage have been evaluated by using visual inspection by a neurologist. Frequency-based spectral analysis, however, was found to be more reliable and accurate than visual analysis (Amodio et al., 1999). Accordingly, the main findings of this study were based on relative power analysis, in which the power of a specific band was divided by the power of all bands. Power ratio analysis was also performed to investigate power in slowing bands compare to other frequency bands. If slowing background activity was identified, theta or delta relative power would be increased correspondingly as well as their involving power ratio.

Although renal failure and diabetes can cause similar forms of cerebral dysfunction, the findings of slowing EEG patterns in ESRD patients with comorbid diabetes is still unclear. Therefore, the primary objective of this study was to investigate slowing EEG patterns in ESRD patients with and without comorbid DM by using quantitative electroencephalogram (QEEG), which was utilized to distinguish patients from healthy controls by comparison to a normative database (Thatcher et al., 2003). Hypotheses were proposed that (1) theta and delta relative power would be higher in ESRD patients with DM (ESRD-DM) than in ESRD patients without DM (ESRD-C) due to the enhancing effect of renal and diabetic complications. Moreover, (2) EEG background activity of each ESRD-DM and ESRD-C patient should have deviated from the mean of normative database due to cerebral complications caused by diabetes or renal failure. These insights may contribute to increase awareness of cerebral complications in ESRD-DM in clinical practice and possibly determine the severity of encephalopathy.

## 2. Materials and Methods

### 2.1. Subjects and Procedures

The experimental procedures described in this cross-sectional case-control study were approved by the Institutional Review Board of Phramongkutklao Hospital with the certificate of approval (COA) number S072h/62 and the Institutional Review Board of Mahidol University with certificate of approval (COA) number MU-CIRB 2020/393.2511. ESRD-C and ESRD-DM who were undergoing kidney replacement therapy, either peritoneal dialysis (PD) or hemodialysis (HD), were recruited. Participants were asked to provide written informed consent before enrollment. They were well-treated with sufficient dialysis (3–4 times a week for HD patients and dialysate drainage every 4 h for PD patients) and were aged more than 40 years. All of them had no history of neurologic or psychiatric disease that could affect electroencephalogram (EEG). Participants were excluded if they had a noisy resting-state EEG signal, which evaluated by test-retest and split-half reliability (Ferguson, 1959). They were also excluded if they had recently taken or had a history of exposure to central nervous system drugs, such as antiepileptic and antidepressant drugs.

After enrollment, subjects were allocated to subgroups based on comorbidity of diabetes. Their gender, age, and dialysis duration were matched to reduce bias of comparison. Subjects were asked to allow their resting-state EEG data to be recorded for 5 min in eyes-closed and eyes-open conditions (overall 10 min) in the daytime (7.00 a.m. to 16.00 p.m.). The experiment was performed a day before subjects underwent hemodialysis (MHD) or 3 h after the last dialysate drainage (PD) session. Due to the COVID-19 pandemic, all subjects needed to complete the experiment while wearing a surgical mask. To reduce muscle and eye movement artifacts during eyes-open EEG recording, subjects were informed that it would be ideal for them to sit upright in an ergonomic chair and stay still with relaxation. Place both legs forward in the most comfortable position. Reduce eye blinking and intentionally look at a black cross-mark that was set at eye level for 5 min. After that, subjects were asked to rest for a minute. Then, eyes-closed EEG data were recorded for five minutes. Eyes-closed and eyes-open EEG conditions were literally different due to the participants' brain arousal stage (Barry and De Blasio, 2017). Eyes-closed EEG was suggested to have more power than eyes-open EEG in all frequency bands, but the patterns of EEG in both eyes-condition were steadily clarified. Therefore, EEG data in this study were investigated in both eyes-closed/open conditions to assure that slowing pathological patterns was consistently observed instead of physiological phenomena solely. To obtain neutral resting-state EEG data, the experimental room was set to be a soundproof. There is closed space with an ivory wall color. The room was temperature-controlled at 25 degrees Celsius and illuminated with sufficient light (300 lux).

An international 10/20 system of electrode placement (19 channels) with reference to the left ear lobule (A1) and ground to the right ear lobule (A2) was applied to record the referential monopolar montage of EEG signals. All titanium nitride electrodes were organized on an elastic cap, which could vary in size to optimally fit the subjects' heads. After wearing the cap, the vertical gap between the electrodes and scalp was filled by conductive gel. Then, a conductive paste-filled gold cup electrode was attached to both ear lobules to ensure that the impedance was less than 5 kΩ. Additionally, two cup electrodes were attached above the right eyebrow (positive electrode) and at the eyelid-cheek junction (reference) to obtain electrooculogram (EOG) for eye blinks. Two more cup electrodes were placed at the left (positive electrode) and right (reference) wrists to record electrocardiogram (ECG). These EOGs and ECGs were intended to be used as EEG artifact-removal guides.

EEG, EOG, and ECG signals were synchronously acquired by connecting all electrodes to a Brain Master Discovery 24E amplifier with a 256 Hz sampling rate and 24-bit accuracy. The signal was applied to a low-pass filter at 80 Hz, monitored, recorded, and stored in a laptop computer by using Brain Master Discovery software. Before recording, the signal offset was monitored on the acquisition screen and optimized until it was less than ten millivolts to maximize signal quality. In the recording period, movement artifacts or electromyograms (EMGs) that affected EEG data were marked on an artifact-record form, which was used to exclude contaminated EEG signals afterward.

### 2.2. EEG Preprocessing, Validation, and Transformation

**Figure 1:**
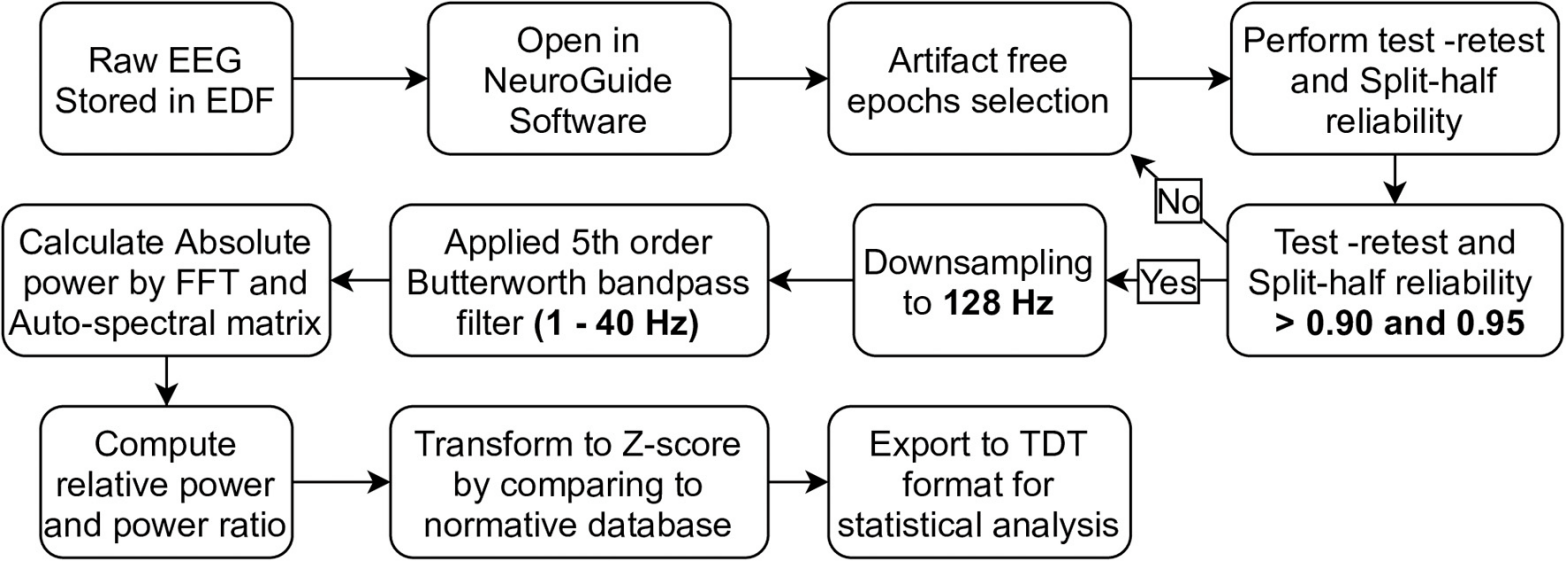
The pipeline of EEG preprocessing, validation, and transformation by using NeuroGuide software were demonstrated in step diagram.

EEGs recorded in the European Data Format (EDF) were preprocessed, validated, and transformed using NeuroGuide software version 2.8.5. These steps were summarized in Figure 1. Artifact-free EEG epochs were selected from both eyes-open and eyes-closed EEG recordings by using EOG, ECG, and artifact-record forms as a guide. Test-retest and split-half reliability were then performed to validate signal homogeneity. Test-retest reliability is the ratio of variance between the first half and the second half of the selected EEG segments, which should have been more than 0.90. Split-half reliability is the ratio of variance between the even and odd seconds of the time-domain EEG, which should have been more than 0.95 as recommended in the software manual. These two statistical methods were used to compare brain state changes and validate the consistency of a measurement (Ferguson, 1959) (as cited in ANI, 2018). It is beneficial to ensure that the selected signal is cleaned and homogeneous enough to represent the brain phenomena underlying it. If the EEG signal is contaminated by noise or other deviated benign artifact, the reliability is decreased. Selected EEGs were then downsampled to 128 Hz, and the 5th order Butterworth bandpass filter was applied at 1–40 Hz. The signals were converted to the frequency domain by fast Fourier transform (FFT), which was used to calculate absolute power, relative power, and the power ratio. In detail, absolute power is the diagonal of the auto-spectral matrix, which was calculated by FFT of the signal multiply by its complex conjugate and divide by the number of frequencies. For relative power, it is the absolute power of specific bands divide by the sum of absolute power of all bands. Power ratio is the absolute power ratio of two specific bands (ANI, 2018). EEG frequency bands involving all calculations were specified as either delta (1.0–4.0 Hz), theta (4.0–8.0 Hz), alpha (8.0–12.0 Hz), beta (12.0–25.0 Hz), or high beta (25.0–30.0 Hz). For the power ratio, delta/theta, delta/alpha, delta/beta, delta/high-beta, theta/alpha, theta/high-beta, alpha/beta, and alpha/high-beta ratios were included in the analysis. To investigate differences in EEG signals from healthy subjects, absolute power, relative power, and the power ratio were then transformed to Z-scores (in range −3.000 to 3.000) based on the normative database (Thatcher et al., 2003), which patients were matched to healthy subjects by age, gender, and recording condition (eyes-closed or eyes-open). Consequently, Z-scores were exported as tab delimiter text (TDT) files to utilize statistical analysis using R programming in RStudio version 1.4.1106.

### 2.3. Statistical Analysis

The distribution of variables was investigated using the Shapiro–Wilk test, and a *p*-value less than 0.05 indicated a significant difference from a normal distribution. In this case, the median and interquartile range (IQR) were used to describe those non-parametric variables [median (IQR)]. For parametric variables, their central values and deviation were represented by the mean and standard deviation (mean ± SD). All Z-scores were transformed according to a normative database, which included healthy people. The mean of those people was represented as zero in the Z-scores so that all Z-scores were included for comparison with zero by using a statistical test. Results were considered to have a significant difference from normal controls if *p*-values were less than 0.05. Statistical analysis began with global comparisons of each frequency band between ESRD-C and ESRD-DM patients in both eyes-closed and eyes-open conditions. The frequency band that was globally significant difference was then encompassed to perform regional comparison. In this case, nineteen EEG channels were reorganized into five regions by representing each region with the central value of the topographical electrodes. These regions are the left frontal (Fp1, F3, and F7), right frontal (Fp2, F4, and F8), frontal (Fp2, F4, F8, Fz, Fp1, F3, and F7), central (C3, Cz, and C4), left temporal (T3 and T5), right temporal (T4 and T6), temporal (T4, T6, T3, and T5), parietal (P3, P4, and Pz), and occipital (O1 and O2) regions. Comparative statistical tests utilized in this study were Student's *t*-test or Mann–Whitney *U*-test depending on variable distribution. A *P*-value less than 0.05 was considered to indicate a significant difference. All statistical analyses were computed by using R programming. In detail, Z-score relative power (9 regions × 5 power bands) and Z-score power ratio (9 regions × 5 ratios) were compared between ESRD-C and ESRD-DM patients (24 subjects in each group).

## 3. Results

**Figure 2:**
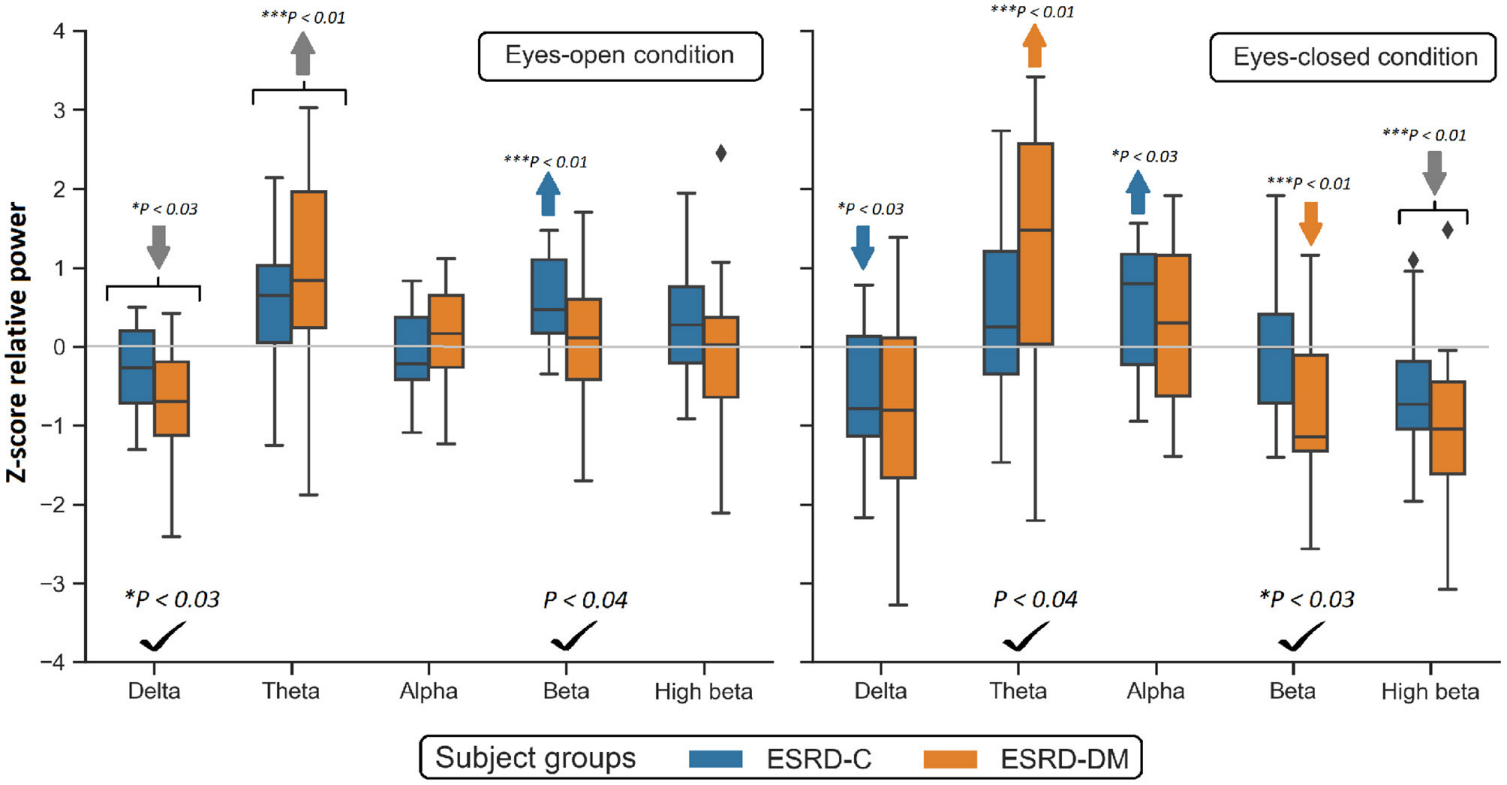
Global Z-scores of relative power in the ESRD-C and ESRD-DM groups was globally found to be different in delta, theta and beta as denoted by check mark. Comparing to healthy control, consistent trend of low delta and high theta was noticeably appeared in both eyes-closed/open condition in ESRD-C (blue) and ESRD-DM (orange) as signified by arrows. *P*-values were calculated by Mann–Whitney *U*-test. The color bar represents IQR, and whiskers were set at an IQR of 1.5. Gray arrow refer to deviation from healthy control in both subject groups.

**Figure 3:**
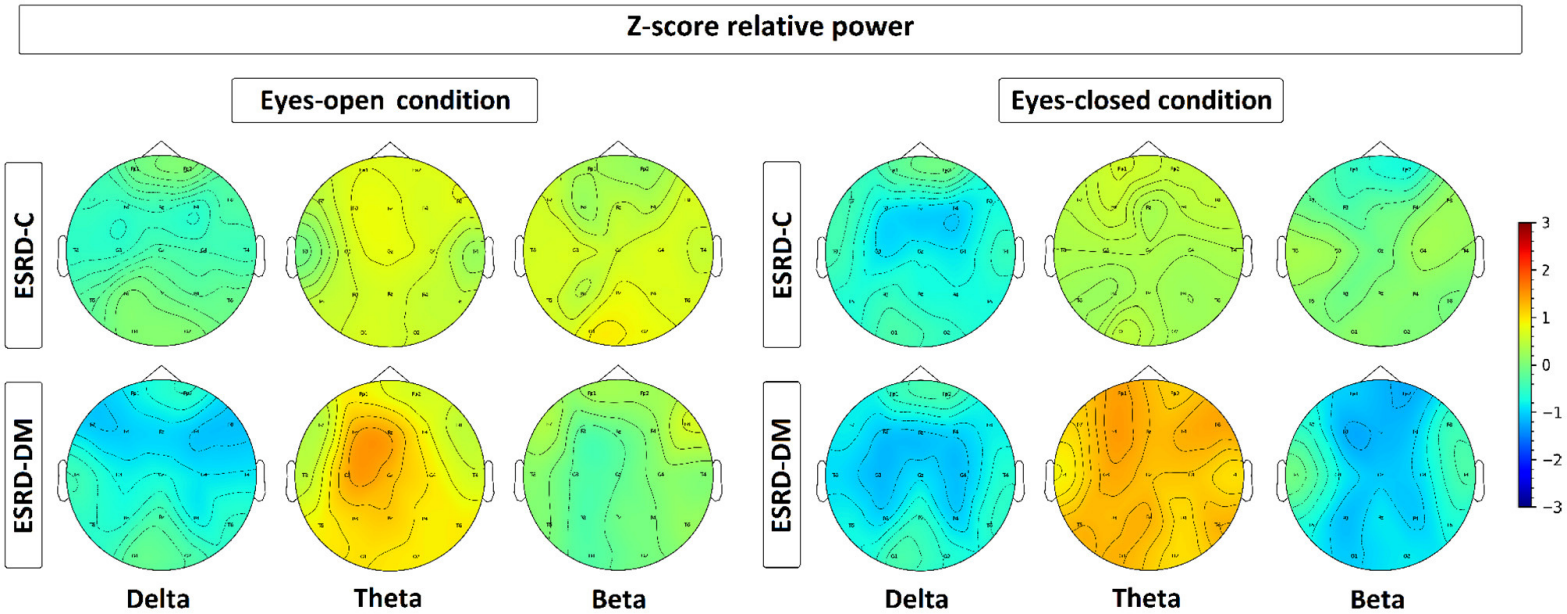
Topographical map depicting the median of Z-score relative power, which is represented by colors varied from −3.00 to 3.00. As seen, the relative power of ESRD-DM denotes high deviation from healthy control in delta, theta, and beta in both eyes-condition. In contrast, delta of ESRD-C in both eyes-condition show solely significant alteration.

**Figure 4:**
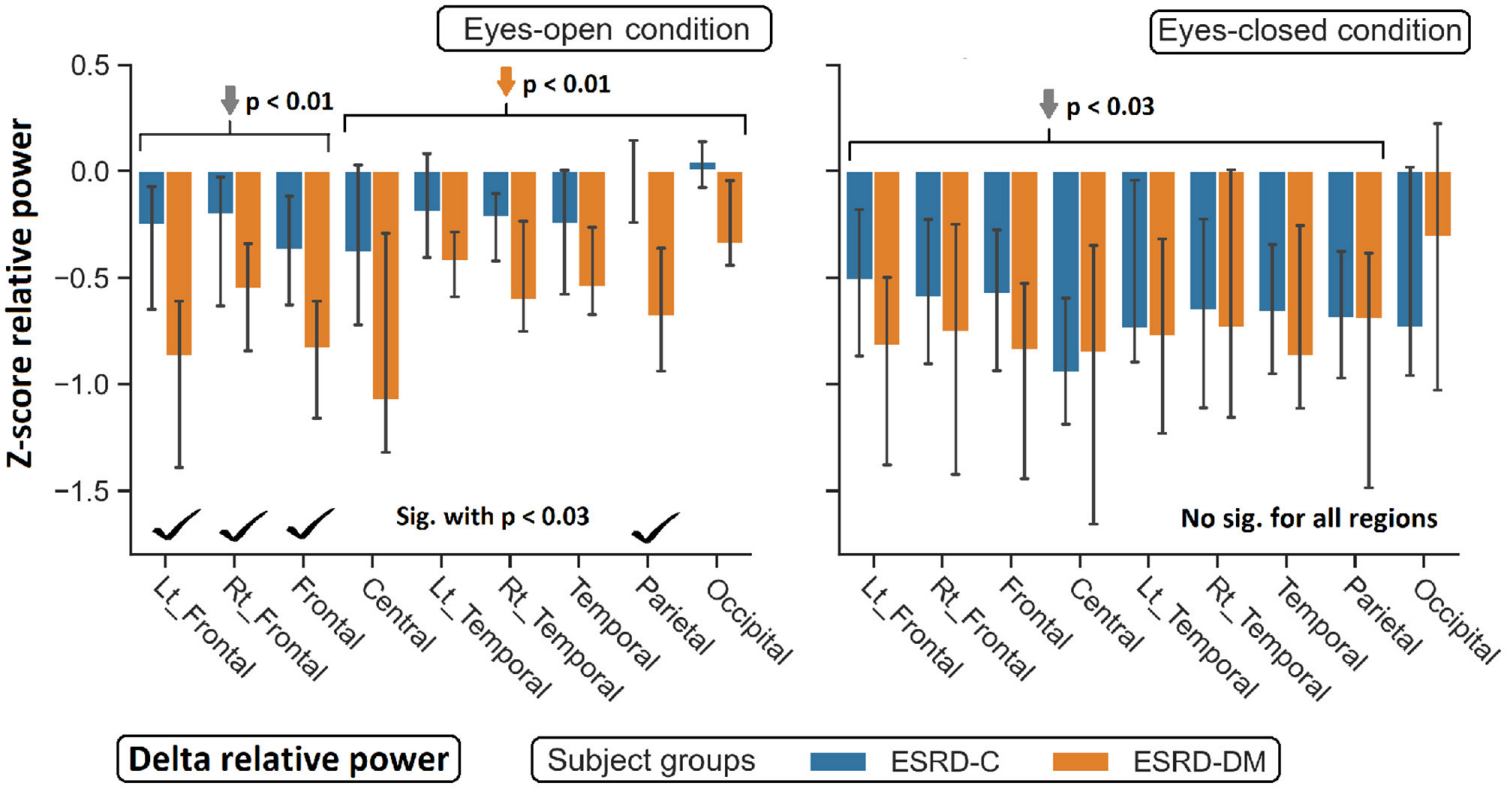
Z-scores of delta relative power in nine topographical regions are demonstrated. The significant difference between eyes-open ESRD-C and ESRD-DM was appeared in frontoparietal regions as signified by check mark. Comparing to healthy control, delta values of ESRD-DM was found lower throughout most regions (orange arrows). *P*-values were calculated by Mann–Whitney *U*-test. The whisker indicates 95 percent confident intervals with median as an estimator. Gray arrows refer to deviation from healthy control in both subject groups.

**Figure 5:**
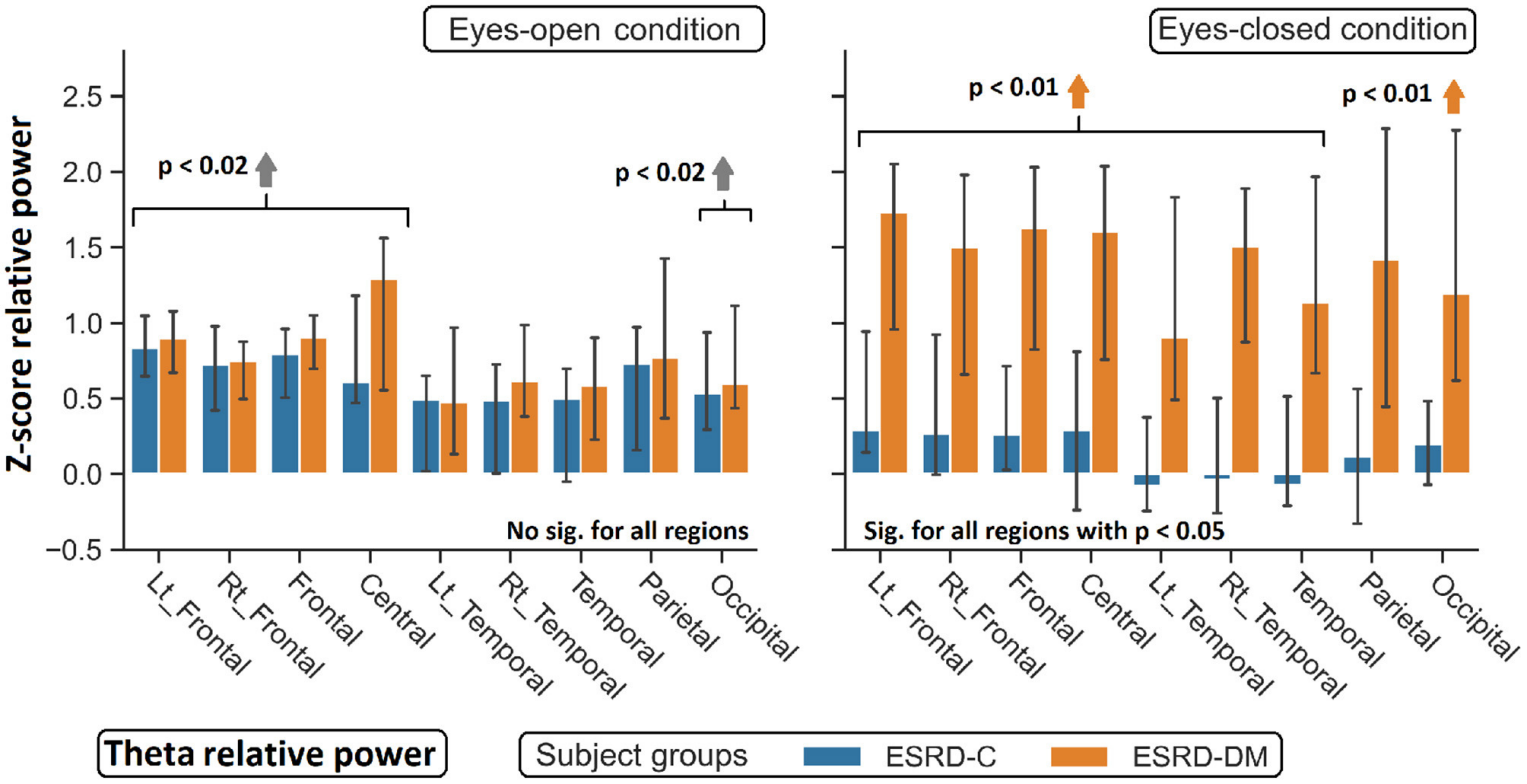
Z-scores of theta relative power in nine topographical regions show significant difference between eyes-closed ESRD-C and ESRD-DM. Comparing to healthy control, higher theta was observed in both subject groups in some regions (gray arrow). Higher theta in eyes-closed ESRD-DM was also found in most regions (orange arrows). *P*-values were calculated by Mann–Whitney *U*-test. The whisker indicates 95 percent confident intervals with median as an estimator.

**Figure 6:**
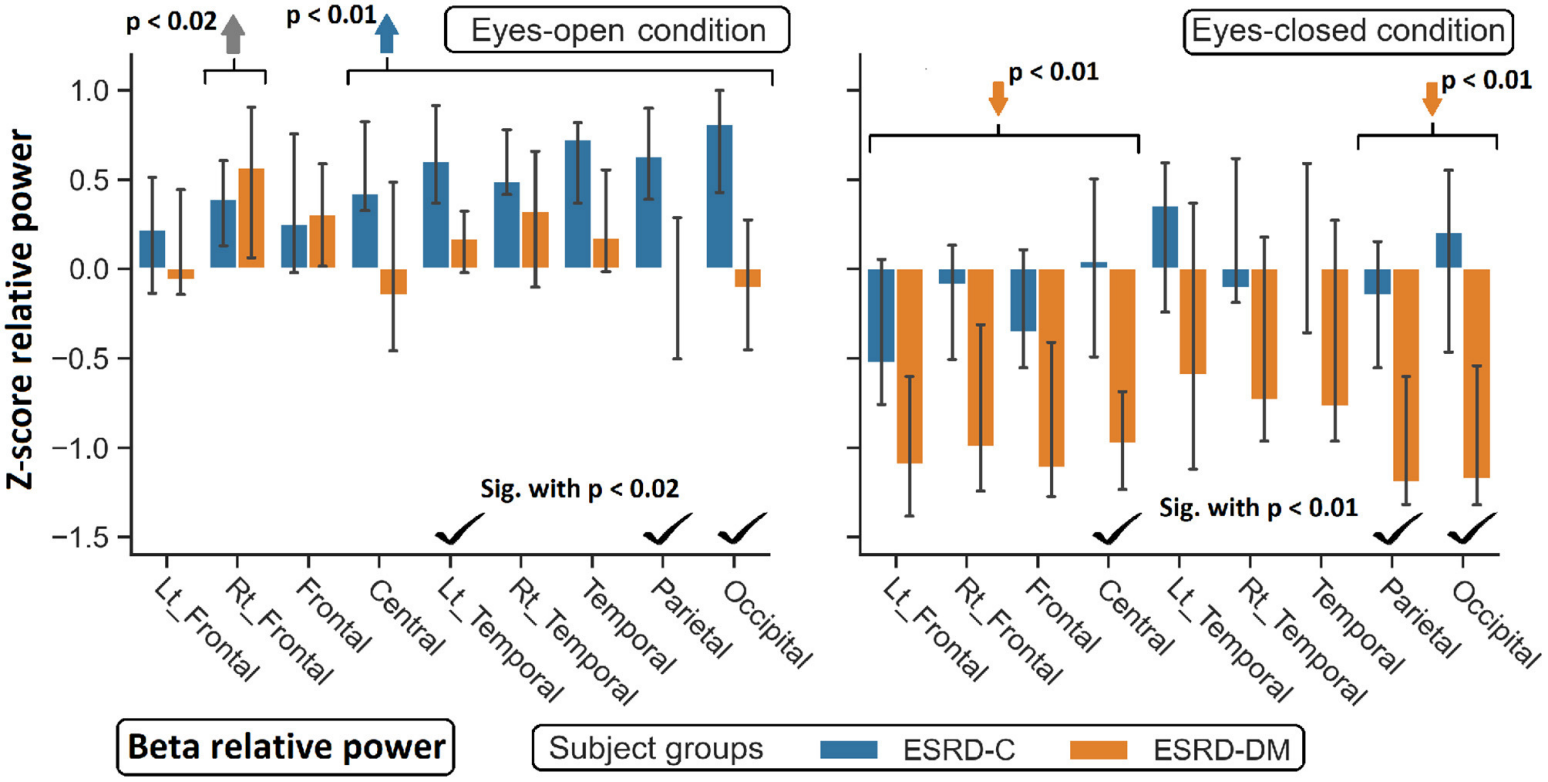
Z-scores of beta relative power were found significant difference between ESRD-C and ESRD-DM as signified by check mark. Comparing to healthy control, ESRD-DM has lower beta in most regions (orange arrows), while ESRD-C has higer beta in most regions (blue arrows). *P*-values were calculated by Mann–Whitney *U*-test. The whisker indicates 95 percent confident intervals with median as an estimator. Gray arrow refer to significant difference from healthy control in both group.

**Figure 7:**
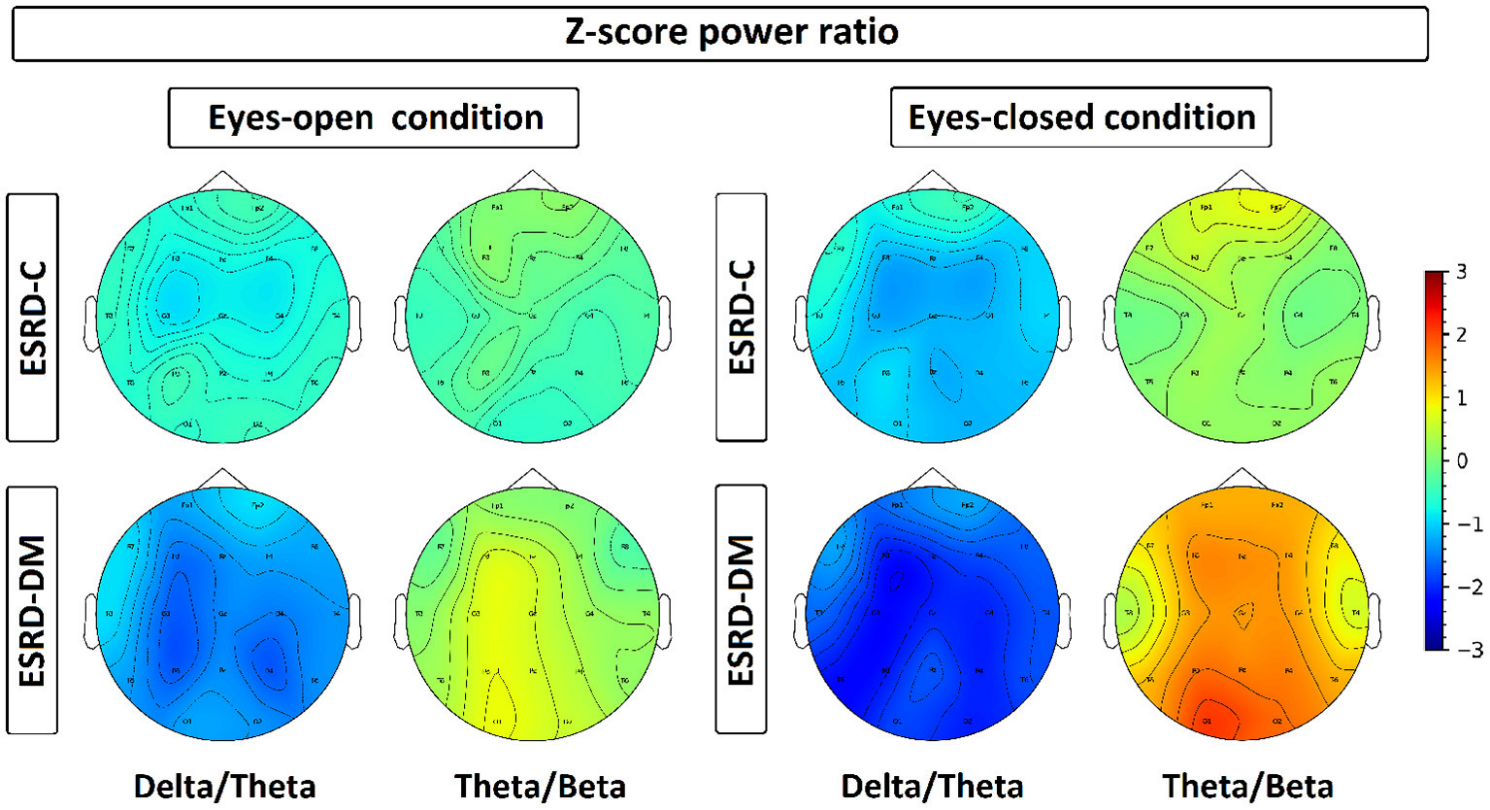
Z-scores of delta/theta and theta/beta ratios have been plotted in a topographical map by representing each channel with its median value, which is represented by color ranged from −3.00 to 3.00. Delta/theta and theta/beta ratio in ESRD-DM were discovered with consistent deviation from healthy control and ESRD-C, especially in eyes-closed condition. On the contrary, these ratio of ESRD-C were rarely deviated from healthy control, except delta/theta in eyes-closed condition.

**Figure 8:**
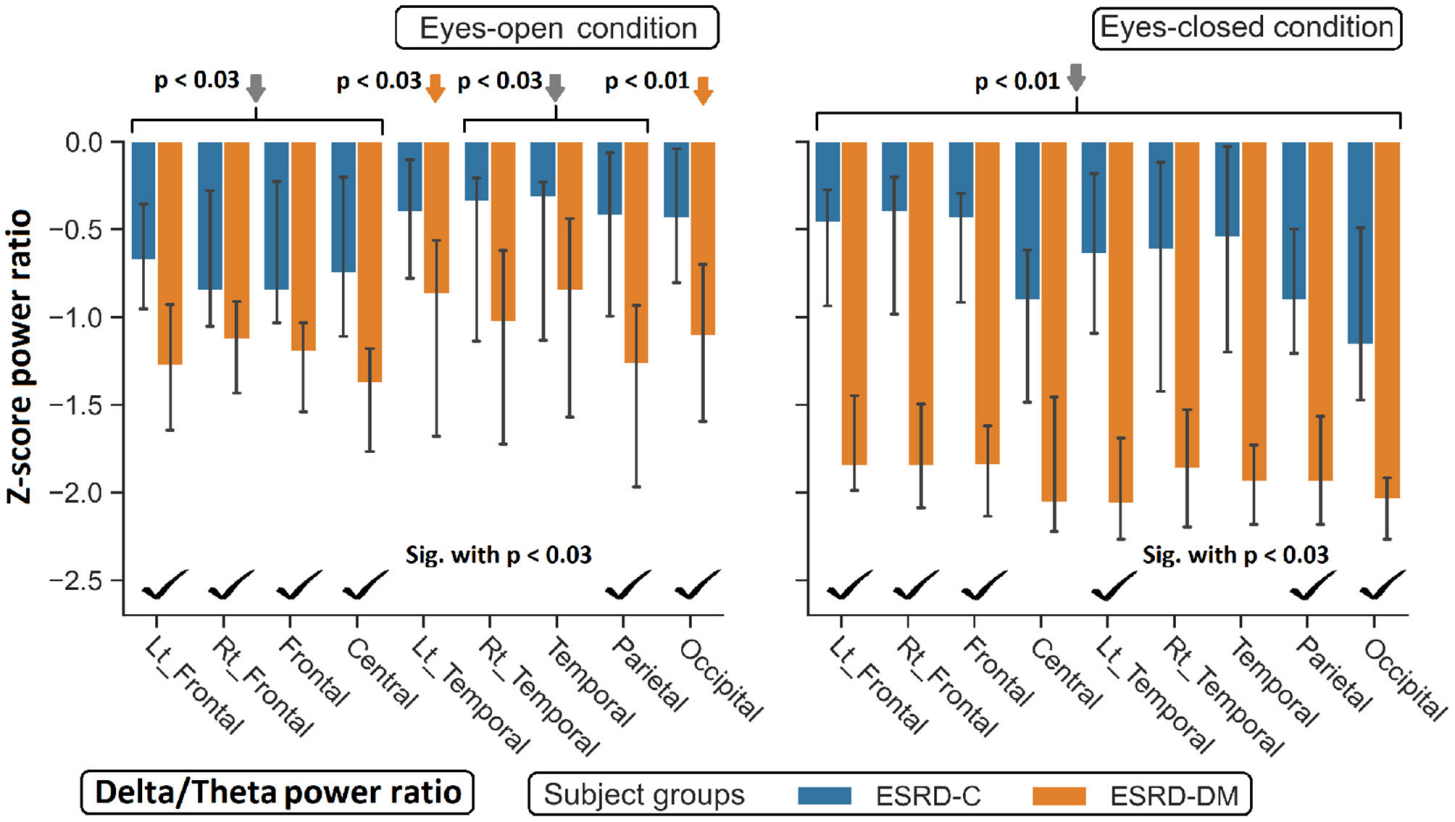
Z-scores of the delta/theta power ratio in most regions between ESRD-C and ESRD-DM were significantly different as signified by check mark. Delta/theta ratio in ESRD-DM globally decreased from healthy control (orange arrows). The same trend was found in ESRD-C. *P*-values were calculated by Mann–Whitney *U*-test. The whisker indicates 95 percent confident intervals with median as an estimator. Gray arrows refer to significant deviation from healthy control in both subject groups.

**Figure 9:**
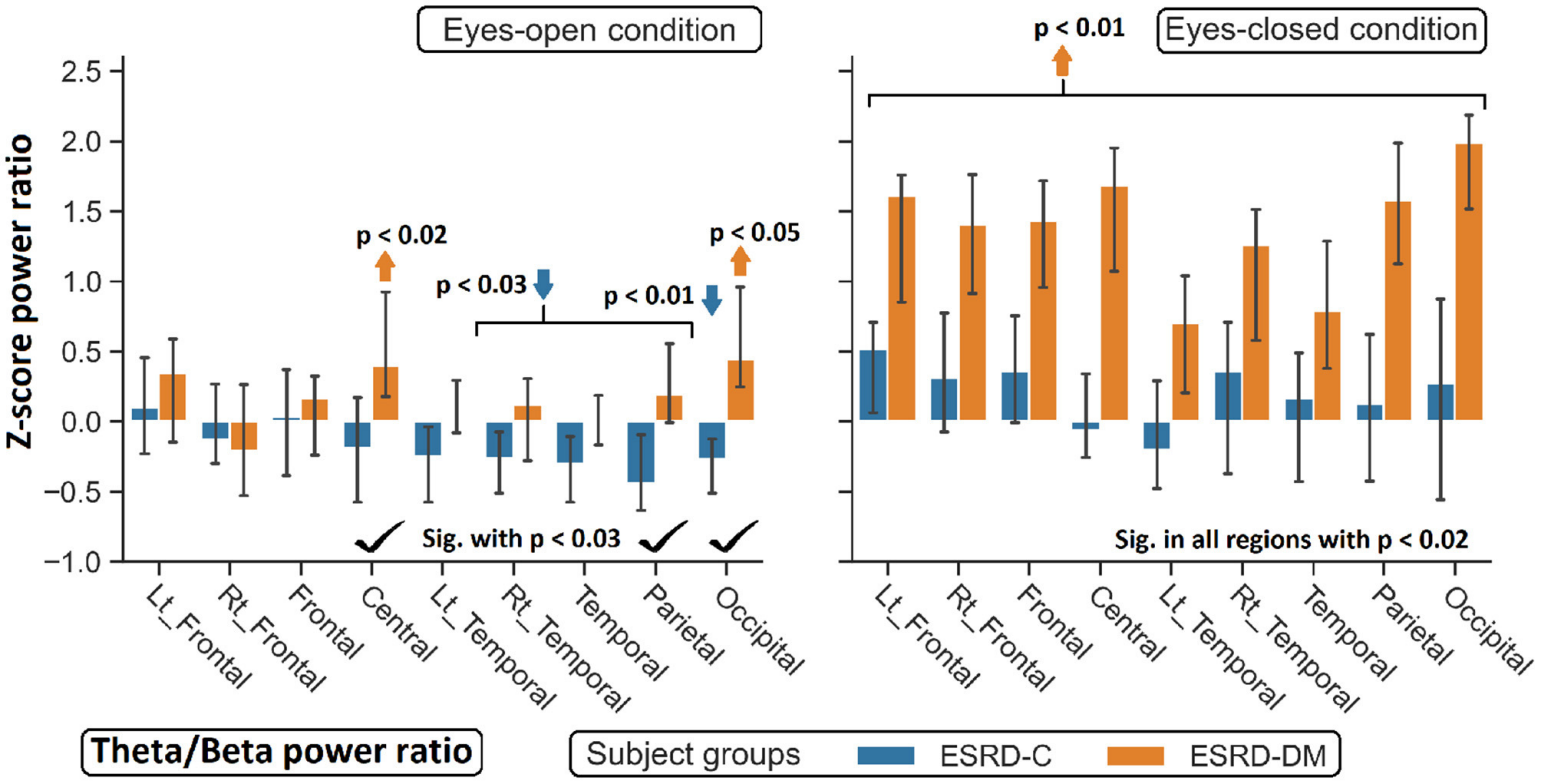
Z-scores of the theta/beta power ratio between ESRD-DM and ESRD-C were globally significant difference in eyes-closed condition, but some significant regions were observed in eyes-open condition as denoted by check mark. Comparing to eyes-closed healthy control, solely theta/beta ratio of ESRD-DM were globally higher (orange arrow). In eyes-open condition, some regions were deviated from healthy control in ESRD-C (blue arrows) and ESRD-DM (orange arrows). *P*-values were calculated by Mann–Whitney *U*-test. The whisker indicates 95 percent confident intervals with median as an estimator.

To investigate the slowing EEG background activity in ESRD-C and ESRD-DM patients, a global comparison of Z-score relative power in five frequency bands was first demonstrated in Figure 2. After that, the Z-score relative power that showed global differences between ESRD-C and ESRD-DM are shown in a topographical map so that the region of significance could be determined (Figures 3–6). Power ratios were also proposed as indices, which could be useful to distinguish between the brain activity of ESRD-C and ESRD-DM patients, as presented in Figures 7–9. Because assumptions of a normal distribution were violated, the median and IQR were used to represent the central value and deviation of each variable. Statistical analysis was performed by using the Mann–Whitney *U*-test. The significance level was set at 0.05, and a *p*-value less than that was considered a significant difference.

### 3.1. Clinical Feature Comparison

In this study, participants were equally recruited into the ESRD-DM (case) and ESRD-C groups (control) as 24 subjects were included in each group. Thirteen and eleven subjects in each group were female and male, respectively. Twelve (F/M - 7/5) ESRD-C subjects were undergoing peritoneal dialysis, and the other 12 (F/M - 6/6) were undergoing machinery hemodialysis. For the ESRD-DM subjects, 11 (F/M - 6/5) were undergoing peritoneal dialysis, and the other 13 (F/M - 7/6) were undergoing machinery hemodialysis. As determined by independent *t*-test, age, and dialysis duration were not significantly different between ESRD-C and ESRD-DM groups, as shown in Table 1, which therefore makes EEG signals comparable between these groups.

**Table 1:** Descriptions of the clinical features of ESRD subjects.

**Descriptions**	**ESRD subjects**		***p*-value**
	**ESRD-C**	**ESRD-DM**	
Number of subjects	24	24	
Sex: female/male	13/11	13/11	
Dialysis types: PD/MHD	12/12	11/13	
Dialysis duration (years)	4.67 ± 3.26	3.78 ± 3.80	0.981
Range of dialysis duration (years)	1.0–13.0	1.5–17.0	
Age (years)	62.00 ± 11.55	61.43 ± 8.47	0.887
Range of age (years)	44–83	47–82	
Hypertension (Yes/No)	15/9	13/11	
Hyperlipidemia (Yes/No)	5/19	7/17	

*Independent t-tests were used to calculate the p-value of age and dialysis duration*.

### 3.2. Global Relative Power Findings

Compared to that in the ESRD-C group, the delta relative power in the eyes-open condition in the ESRD-DM group was significantly lower (*p* = 0.025); however, the theta value in the eyes-closed condition was found to be higher (*p* = 0.038). The beta relative power of the ESRD-DM group was lower in both the eyes-open and eyes-closed conditions, with *p* = 0.032 and 0.022, respectively. Also, the delta value in the eyes-open condition, theta value in the eyes-closed condition, and beta value in both conditions were found to be significantly different between ESRD-C and ESRD-DM groups, as presented in Figure 2. Thus, further analysis results encompass solely delta, theta, and beta values.

In comparison to the zero-mean normative database, delta relative power in the ESRD-C (*p* = 0.022) and ESRD-DM (*p* = 0.001) groups in the eyes-open condition was lower than that in healthy control group and delta relative power in the eyes-closed condition in the ESRD-C group (*p* = 0.022). Theta power in the eyes-open condition in the ESRD-C (*p* = 0.004) and ESRD-DM (*p* = 0.001) groups were significantly higher than that in healthy control group and that in the eyes-closed condition in the ESRD-DM group (*p* = 0.004). Beta power in the eyes-open condition in the ESRD-C group (*p* < 0.001) and the eyes-closed condition in the ESRD-DM group (*p* = 0.001) were also significantly higher than the beta power measured in other groups, as demonstrated in Figure 2.

### 3.3. Topographical Findings in Delta, Theta, and Beta Relative Power

As seen in Figure 3, the eyes-closed delta values and eyes-open theta values were not significantly different between ESRD-C and ESRD-DM groups; however, it is worth mentioning that eyes-closed delta values in the ESRD-C and ESRD-DM groups, except in the occipital regions in both groups, were globally lower than those of the healthy control group (*p* < 0.03) (Figure 4). The eyes-open theta values of both ESRD-C and ESRD-DM groups were generally higher than those of the healthy control group in most regions (*p* < 0.02), except the parieto-temporal regions of the ESRD-C group (Figure 5).

As extended from global relative power findings, the eyes-open delta values of the ESRD-DM group was considerably lower than that of the ESRD-C group in the fronto-parietal region with *p* < 0.03 (Figure 4), and the eyes-closed theta values of the ESRD-DM group was greater than those in the ESRD-C group in all regions with *p* < 0.05 (Figure 5). Beta values in both conditions in the ESRD-DM group were lower than those in the ESRD-C group in the parieto-occipital regions with *p* < 0.01; in addition, the eyes-open beta values in the left temporal region (*p* = 0.017) and eyes-closed beta values in the central region (*p* = 0.006) of the ESRD-DM group were also lower than those in the ESRD-C group (Figure 6).

Compared to the values in the healthy control group, the eyes-open delta values of the ESRD-DM group was globally lower in all regions with *p* < 0.01, but delta values in the eyes-open ESRD-C group was only lower in frontal regions with *p* < 0.03 (Figure 5). The eyes-closed theta values in the ESRD-DM group were higher in most regions with *p* < 0.01, except the parietal region, which was not significantly different (Figure 6). Beta values in the eyes-open condition in the ESRD-C group was higher in most regions with *p* < 0.01, except the left frontal region, which led to no significant difference in the overall frontal region. Notably, values in the right frontal region were still significantly higher, with *p* = 0.004. Beta values in the eyes-closed condition in the ESRD-C group was, however, not different from those in the healthy control group. For beta values in the ESRD-DM group, eyes-open beta values were only higher in the right frontal region with *p* = 0.011, but eyes-closed beta values were lower in most regions with *p* < 0.01, except in the left and right temporal regions, which did not have significantly different values (Figure 7).

### 3.4. Topographical Findings in the Delta/Theta and Theta/Beta Indexes

Findings in regional relative power clearly identified patterns in both the eyes-closed and eyes-open conditions in the ESRD-DM group; these patterns were low delta, high theta, and low beta power. Therefore, Z-score delta/theta and theta/beta ratios were additionally analyzed to identify more features that could be used to distinguish brain activity between the participants in the ESRD-C and ESRD-DM groups. As seen in Figure 7, the delta/theta ratio in both conditions in the ESRD-DM group was generally lower than that in ESRD-C group in the fronto-parieto-occipital regions, with *p* < 0.03. In addition, values in the eyes-open central region and eyes-closed left temporal region were significantly different, with *p* < 0.03 (Figure 8). The theta/beta ratio in the eyes-closed condition was globally different between ESRD-C and ESRD-DM groups in all channels, with *p* < 0.02. In addition, the theta/beta ratio in the eyes-open condition in the ESRD-DM group was significantly greater than that in the ESRD-C group in the centro-parieto-occipital regions, with *p* < 0.03 (Figure 9).

Compared to that in the healthy control group, the delta/theta ratio was globally lower in both subject groups and both conditions with *p* < 0.03, except in the left temporal and occipital regions in eyes-open condition in the ESRD-C group, which showed no significance (Figure 8). The theta/beta ratio in the eyes-closed condition in the ESRD-DM group was significantly greater than that of the healthy group in all regions, with *p* < 0.01. The values in the centro-occipital region in the eyes-open condition in the ESRD-DM group were also higher than those in the ESRD-C and healthy control groups, with *p* = 0.004 and *p* < 0.05, respectively. In contrast, the theta/beta ratio in the eyes-open condition in the ESRD-C group was lower than that in the healthy control group in the right temporal and parieto-occipital regions, with *p* < 0.03 (Figure 9).

## 4. Discussion

### 4.1. Main Results

Hypotheses of this study were that (1) theta and delta relative power would be higher in ESRD-DM than in ESRD-C due to the enhancing effect of renal and diabetic complications. Moreover, (2) EEG background activity of each ESRD-DM and ESRD-C patient should have deviated from the mean of normative database due to cerebral complications caused by diabetes or renal failure.

As the thorough analysis and all demonstrated results, differences in EEG background activity between the ESRD-DM and ESRD-C group revealed that the delta relative power of the frontoparietal regions in the eyes-open ESRD-DM group was lower than that in the ESRD-C group, which violated the first hypothesis of this study. Also, generalized theta relative power in the eyes-closed ESRD-DM group was higher than that in the ESRD-C group. Moreover, the beta power of the parieto-occipital regions in the both eyes-closed and eyes-open conditions in the ESRD-DM group was lower than that in the ESRD-C group. In addition, eyes-open beta values in the left temporal regions and eyes-closed beta values in the central regions were also lower. These low-high-low patterns *(EO frontoparietal delta: ESRD-DM*<*ESRD-C, EC generalized theta: ESRD-DM* > *ESRD-C, and EO-EC parieto-occipital beta: ESRD-DM*<*ESRD-C)* in the ESRD-DM group were substantially increased compared to those in the ESRD-C group. Among these patterns, only the eyes-open frontal delta value was able to be used as a classifier of healthy controls, ESRD-C patients, and ESRD-DM patients. The relative power of the eyes-open frontal delta value in ESRD-DM patients was the highest, and that in ESRD-C patients was higher than that in healthy controls *(EO-frontal delta: ESRD-DM patients* > *ESRD-C patients* > *healthy controls)*. Noticeably, frontal delta and frontal theta relative power in both conditions and subject groups tended to be lower and higher than those in the healthy control group, but the difference between ESRD-C and ESRD-DM groups was not significant in some regions.

Although the parieto-occipital beta relative power was clearly different between ESRD-C and ESRD-DM patients in both eyes-closed/open conditions, this feature was not optimal for use as a classifier for healthy controls because this feature in the eyes-open condition in the ESRD-DM group and the eyes-closed condition in the ESRD-C group was not different from that in healthy controls. Therefore, these patterns of relative power inspired an analysis of delta/theta and theta/beta power ratios, which were expected to show clear differences among ESRD-DM patients, ESRD-C patients, and healthy controls. As demonstrated previously, the frontoparietal delta/theta power ratio in both eye conditions was suitable for application as a classifier *(EO-EC frontoparietal delta/theta: ESRD-DM patients*<*ESRD-C patients*<*healthy controls)*; however, the theta/beta ratio may not be suited to classify ESRD-C patients from healthy controls, but the pattern of theta/beta ratio values appeared to be noticeably different between ESRD-C and ESRD-DM patients in the eyes-closed condition *(EC generalized theta/beta: ESRD-DM patients* > *ESRD-C patients* = *healthy controls)*. Notably, zero Z-scores from a normative database were represented as healthy control median.

### 4.2. Clinical Interpretation

Z-score analysis results in this study should be interpreted and considered with caution. Because differences in Z-scores among subjects were not specific to brain abnormalities, these deviations may indicate the possibility of brain alterations arising from clinical complications. For example, cerebral dysfunction is a cause of background slowing of EEG signals in the delta and theta frequency ranges, but these slowing patterns do not always refer to cerebral dysfunction (Louis and Frey, 2016). In this study, uremic and diabetic encephalopathy, which may lead to a short attention span and cognitive impairment, were thought to be the cause of brain functional alterations. Even though participants had been asked to have their EEG signals recorded 1 day before hemodialysis or 3 h after dialysate was drained, some background activity found in ESRD-C patients marginally deviated from that of healthy controls. In contrast, most background activity of ESRD-DM patients varied considerably from that of healthy controls and ESRD-C patients, which may indicate higher risk of brain complications in ESRD-DM.

In this study, the comorbid effects of both uremic and diabetic encephalopathy in ESRD-DM patients were suggested as a cause of this variation. Relative power and power ratio findings showed that higher parieto-occipital beta in the eyes-open ESRD-C group may have been the effect of concentration during focusing on cross-mark. For the ESRD-DM group, this result may have been related to the focused attention problem because the beta during concentration was not higher than resting-state beta activity. This concept was also supported by decreased frontoparietal delta, increased fronto-centro-occipital theta, and an increased theta/beta ratio of ESRD-DM patients compared to healthy controls in both eyes-closed/open conditions. More specifically, delta oscillation was suggested that it is associated with sensory interference inhibitor, which normal delta activity was found to be higher during mental tasks (Harmony, 2013). The theta/beta ratio was reported as a marker of cognitive processing capacity, which can be used to investigate attention deficit hyperactivity disease (AD/HD). It has been shown that an increased theta/beta ratio may lead to decreased cognitive capacity (Lansbergen et al., 2011; Kropotov, 2016; Clarke et al., 2019; Picken et al., 2019), which indicates the difficulty of maintaining focused attention. Moreover, theta during focused attention was found to be lower in all channels compared to resting-state EEG (Lim et al., 2019). Therefore, delta, theta, and beta patterns observed in ESRD-DM may refer to attention deficit caused by aforementioned comorbidity.

Attentional impairment may be the cause of cerebral deterioration in both ESRD-DM (most) and ESRD-C (some) patients as observed by theta/beta ratio. Encephalopathic symptoms such as cognitive impairment may be another cause of cerebral deterioration. Theta relative power can also refer to the severity of cognitive impairment, and it has been suggested that the progression from mild cognitive impairment to Alzheimer's disease can be determined by increased theta relative power (Jelic et al., 2000; Moretti et al., 2007). The frontoparietal delta/theta ratio of ESRD-DM and ESRD-C was found to be less than that of healthy controls in both eyes-closed/open conditions. This ratio may be the optimal index to identify the severity of encephalopathy. The literature supports the claim that patients with metabolic and endocrine encephalopathy exhibit an increased theta/delta ratio compared to healthy controls (decreased delta/theta). The causes of encephalopathy include hypoglycemia, hyperglycemia, vitamin deficiency, electrolyte derangement, hormonal disorder, and accumulation of uremic toxin (Faigle et al., 2013; Hamed, 2019). Therefore, greater severity due to the comorbidity of diabetes and renal failure may be observed by using the delta/theta ratio, which suggests that the more severe encephalopathy was, the lower the delta/theta ratio was.

### 4.3. Limitations

The relative power and power ratio of ESRD-C and ESRD-DM patients were transformed to Z-scores by using a normative database, which was based on data recorded from healthy subjects in the resting stage. A comparison of Z-scores to zero was performed to determine the deviation of patients from healthy controls, where zero indicates the mean or median of the relative power of age-matched healthy controls. This was considered a limitation of providing standard error for healthy controls that may result in uncertainty in the interpretation of the results. Additionally, all Z-scores could only be obtained from specific licensed software, which limits the reproducibility of this research. Finally, the fact that the EEG signals were not specific to a certain abnormality needs to be considered; for example, multifactorial pathogenesis can generate the same slowing EEG patterns (Louis and Frey, 2016). Therefore, the presented findings in this study only suggests the possible cause of attentional and cognitive impairment.

## 5. Conclusion

Based on the QEEG background activity findings in this study, clear signs of attentional and cognitive impairment were found in ESRD-DM patients, and slight indications were found in ESRD-C patients because of encephalopathic effects. Those sign of brain alteration indicated beta, delta, and theta relative power, which can be more noticeable as observed in delta/theta and theta/beta ratio with the variation of electrode regions and eyes-conditions. Particularly in ESRD-DM, theta/beta ratio, an index of attention deficit, was globally determined in eyes-closed condition; in addition, delta/theta ratio as an index of encephalopathy was also clarified in both eyes-closed/open condition. Therefore, cerebral complications in end-stage renal disease comorbid with diabetes are recommended to be greater concern in clinical practice. Moreover, this study suggested that the frontoparietal delta/theta ratio may be a possible feature of EEG background activity that could determine the severity of encephalopathic comorbidity.

## Data Availability Statement

The raw data supporting the conclusions of this article will be made available by the authors, without undue reservation.

## Ethics Statement

The studies involving human participants were reviewed and approved by the Institutional Review Board of Phramongkutklao Hospital with the certificate of approval (COA) number S072h/62 and the Institutional Review Board of Mahidol University with the certificate of approval (COA) number MU-CIRB 2020/393.2511. The patients/participants provided their written informed consent to participate in this study.

## Author Contributions

TJ, PT, OS, and YW contributed to conception and design of the study. TJ performed the experiment and statistical analysis. TJ and YW wrote the first draft of the manuscript. All authors contributed to manuscript revision, read, and approved the submitted version.

## Conflict of Interest

The authors declare that this study received funding from Thai Union Group PCL. The funder was not involved in the study design, collection, analysis, interpretation of data, the writing of this article or the decision to submit it for publication.

## Publisher's Note

All claims expressed in this article are solely those of the authors and do not necessarily represent those of their affiliated organizations, or those of the publisher, the editors and the reviewers. Any product that may be evaluated in this article, or claim that may be made by its manufacturer, is not guaranteed or endorsed by the publisher.
